# Supplementary material for: Gestational breast cancer in New South Wales: A population-based linkage study of incidence, management, and outcomes
Source: PLoS One. 2021 Jan 22;16(1):e0245493. doi: 10.1371/journal.pone.0245493 (PMC7822528; doi:10.1371/journal.pone.0245493)
Supplement: S1 Table — (DOCX) [file pone.0245493.s001.docx]

**S1 Table:** Timing of diagnosis and stage of cancer by gestational age at birth for the 93 women who gave birth by induction of labour or prelabour CS.

|  | **Preterm<37 weeks** | **Term =>37 weeks** | **OR (95% CI)** |
| --- | --- | --- | --- |
|  | **N (%)*** | **N (%)*** |  |
| **Timing of diagnosis** |  |  |  |
| 1st trimester | 7(70) | 3(30) | Reference |
| 2nd trimester | 19(59.4) | 13(40.6) | 0.63 (0.14-2.88) |
| 3rd trimester* | 22(56.4) | 17(43.6) | 0.55 (0.12-2.47) |
| **Cancer stage** |  |  |  |
| Stage 1 | 14(51.9) | 13(48.1) | Reference |
| Stages 2-3 | 31(57.4) | 23(42.6) | 1.25 (0.49-3.17) |
| Stages 4 | 1(16.7) | 5(83.3) | 0.19 (0.02-1.81) |
| Not stated** | 2(33.3) | 4(66.7) |  |
| *For women who were diagnosed before 37 weeks only, **Not included in the analysis | | | |
