# Supplementary material for: Gestational breast cancer in New South Wales: A population-based linkage study of incidence, management, and outcomes
Source: PLoS One. 2021 Jan 22;16(1):e0245493. doi: 10.1371/journal.pone.0245493 (PMC7822528; doi:10.1371/journal.pone.0245493)
Supplement: S2 Table — (DOCX) [file pone.0245493.s002.docx]

**S2 Table:** Characteristics of singleton preterm babies by cancer status.

| **Outcome** | **Breast cancer** | **No cancer** |
| --- | --- | --- |
| **Gender of baby** | | |
| Male | 29(54.7) | 55779(54.8) |
| Female | 24(45.3) | 45889(45.1) |
| Indeterminate | 0(0) | 117(0.1) |
| Not stated | 0(0) | 49(0) |
| **Gestational age** | | |
| ≤33 weeks | 18(34.0) | 30854(30.3) |
| 34-36 weeks | 35(66.0) | 70980(69.7) |
| **Birthweight*** | | |
| <2000 | 7(13.2) | 23289(24.7) |
| 2000 to <2500 | 22(41.5) | 25206(26.7) |
| 2500 to <3000 | 20(37.7) | 29512(31.3) |
| 3000 or over | 4(7.5) | 16120(17.1) |
| Not stated | 0(0.0) | 120(0.1) |
| **Discharge status** | | |
| Discharged | 47(88.7) | 72942(71.6) |
| Stillborn | 0(0) | 7587(7.5) |
| Neonatal death | 0(0) | 3161(3.1) |
| Transferred | 6(11.3) | 17862(17.5) |
| Not stated | 0(0) | 282(0.3) |
| **Timing of maternal cancer diagnosis** | | |
| 1st trimester | 10(18.9) | NA |
| 2nd trimester | 20(37.7) | NA |
| 3rd trimester | 23(43.4) | NA |
| **Stage of maternal cancer** | | |
| Stage 1 | 17(32.1) | NA |
| Stages 2-3 | 32(60.4) | NA |
| Stages 4 | 2(3.8) | NA |

*Excluding stillbirth
