# Supplementary material for: Gestational breast cancer in New South Wales: A population-based linkage study of incidence, management, and outcomes
Source: PLoS One. 2021 Jan 22;16(1):e0245493. doi: 10.1371/journal.pone.0245493 (PMC7822528; doi:10.1371/journal.pone.0245493)
Supplement: S3 Table — (DOCX) [file pone.0245493.s003.docx]

**S3 Table:** Regression model of Table 1

| **Dependent variable: Breast Cancer During Pregnancy** | | | | | | | | |
| --- | --- | --- | --- | --- | --- | --- | --- | --- |
|  | B | S.E. | Wald | df | Sig. | Exp(B) | 95% C.I.for EXP(B) | |
|  |  |  |  |  |  |  | Lower | Upper |
| Country of birth | 0.156 | 0.218 | 0.514 | 1 | 0.473 | 1.169 | 0.763 | 1.791 |
| Maternal age | 1.817 | 0.209 | 75.532 | 1 | 0.000 | 6.155 | 4.086 | 9.273 |
| Parity | 0.078 | 0.221 | 0.126 | 1 | 0.723 | 1.082 | 0.701 | 1.668 |
| Plurality | -0.654 | 1.005 | 0.423 | 1 | 0.516 | 0.520 | 0.073 | 3.731 |
| Previous CS | -0.026 | 0.278 | 0.009 | 1 | 0.926 | 0.975 | 0.565 | 1.681 |
| Smoking during pregnancy | -1.245 | 0.514 | 5.862 | 1 | 0.015 | 0.288 | 0.105 | 0.789 |
| Pre-existing hypertension | 0.889 | 0.587 | 2.296 | 1 | 0.130 | 2.433 | 0.770 | 7.686 |
| Remoteness |  |  | 0.856 | 3 | 0.836 |  |  |  |
| Inner Regional | -0.006 | 0.288 | 0.000 | 1 | 0.985 | 0.994 | 0.565 | 1.749 |
| Outer Regional | -0.305 | 0.592 | 0.266 | 1 | 0.606 | 0.737 | 0.231 | 2.351 |
| Remote/very remote | 0.759 | 1.010 | 0.565 | 1 | 0.452 | 2.137 | 0.295 | 15.466 |
| Constant | -10.363 | 0.244 | 1803.626 | 1 | 0.000 | 0.000 |  |  |

| **Hosmer and Lemeshow Test** | | | |
| --- | --- | --- | --- |
| Step | Chi-square | df | Sig. |
| 1 | 7.240 | 8 | 0.511 |
