# Supplementary material for: Gestational breast cancer in New South Wales: A population-based linkage study of incidence, management, and outcomes
Source: PLoS One. 2021 Jan 22;16(1):e0245493. doi: 10.1371/journal.pone.0245493 (PMC7822528; doi:10.1371/journal.pone.0245493)
Supplement: S5 Table — (DOCX) [file pone.0245493.s005.docx]

**S5 Table:** Regression models of Table 3.

| **Model 1: Dependent variable - Sex of baby** | | | | | | | | |
| --- | --- | --- | --- | --- | --- | --- | --- | --- |
|  | B | S.E. | Wald | df | Sig. | Exp(B) | 95% C.I.for EXP(B) | |
|  |  |  |  |  |  |  | Lower | Upper |
| Breast Cancer During Pregnancy | 0.092 | 0.183 | 0.256 | 1 | 0.613 | 1.097 | 0.767 | 1.569 |
| Country of birth | 0.007 | 0.004 | 3.327 | 1 | 0.068 | 1.007 | 1 | 1.014 |
| Maternal age | 0.002 | 0.004 | 0.293 | 1 | 0.588 | 1.002 | 0.994 | 1.01 |
| Parity | 0.004 | 0.003 | 1.763 | 1 | 0.184 | 1.004 | 0.998 | 1.01 |
| Smoking during pregnancy | -0.003 | 0.004 | 0.529 | 1 | 0.467 | 0.997 | 0.988 | 1.005 |
| Pre-existing hypertension | -0.033 | 0.016 | 4.035 | 1 | 0.045 | 0.968 | 0.937 | 0.999 |
| Remoteness |  |  | 1.916 | 3 | 0.590 |  |  |  |
| Inner Regional | 0.001 | 0.004 | 0.064 | 1 | 0.800 | 1.001 | 0.993 | 1.009 |
| Outer Regional | 0 | 0.007 | 0.001 | 1 | 0.972 | 1 | 0.986 | 1.014 |
| Remote/very remote | 0.025 | 0.018 | 1.888 | 1 | 0.169 | 1.025 | 0.989 | 1.063 |
| Hospital sector | -0.005 | 0.004 | 1.664 | 1 | 0.197 | 0.995 | 0.987 | 1.003 |
| Constant | -0.067 | 0.006 | 138.973 | 1 | 0.000 | 0.935 |  |  |

| **Model 2: Dependent variable - Preterm birth** | | | | | | | | |
| --- | --- | --- | --- | --- | --- | --- | --- | --- |
|  | B | S.E. | Wald | df | Sig. | Exp(B) | 95% C.I.for EXP(B) | |
|  |  |  |  |  |  |  | Lower | Upper |
| Breast Cancer During Pregnancy | 2.578 | 0.186 | 191.871 | 1 | 0.000 | 13.167 | 9.143 | 18.962 |
| Country of birth | 0.099 | 0.008 | 160.26 | 1 | 0.000 | 1.104 | 1.087 | 1.121 |
| Maternal age | 0.282 | 0.008 | 1181.864 | 1 | 0.000 | 1.326 | 1.305 | 1.348 |
| Parity | -0.243 | 0.007 | 1312.25 | 1 | 0.000 | 0.784 | 0.774 | 0.795 |
| Smoking during pregnancy | 0.503 | 0.008 | 3773.522 | 1 | 0.000 | 1.654 | 1.628 | 1.681 |
| Pre-existing hypertension | 0.978 | 0.024 | 1706.515 | 1 | 0.000 | 2.66 | 2.54 | 2.787 |
| Remoteness |  |  | 175.054 | 3 | 0.000 |  |  |  |
| Inner Regional | -0.092 | 0.009 | 103.856 | 1 | 0.000 | 0.912 | 0.896 | 0.929 |
| Outer Regional | -0.153 | 0.015 | 98.381 | 1 | 0.000 | 0.858 | 0.833 | 0.885 |
| Remote/very remote | -0.004 | 0.036 | 0.012 | 1 | 0.914 | 0.996 | 0.928 | 1.07 |
| Hospital sector | -0.444 | 0.009 | 2212.526 | 1 | 0.000 | 0.641 | 0.63 | 0.653 |
| Constant | -2.787 | 0.007 | 138124.5 | 1 | 0.000 | 0.062 |  |  |

| **Model 3: Dependent variable - Small for gestation** | | | | | | | | |
| --- | --- | --- | --- | --- | --- | --- | --- | --- |
|  | B | S.E. | Wald | df | Sig. | Exp(B) | 95% C.I.for EXP(B) | |
|  |  |  |  |  |  |  | Lower | Upper |
| Breast Cancer During Pregnancy | 0.165 | 0.308 | 0.287 | 1 | 0.592 | 1.179 | 0.645 | 2.155 |
| Country of birth | -0.425 | 0.006 | 5278.947 | 1 | 0.000 | 0.654 | 0.646 | 0.661 |
| Maternal age | -0.007 | 0.007 | 1.085 | 1 | 0.298 | 0.993 | 0.979 | 1.006 |
| Parity | -0.565 | 0.005 | 11737.47 | 1 | 0.000 | 0.568 | 0.562 | 0.574 |
| Smoking during pregnancy | 0.947 | 0.006 | 22964.83 | 1 | 0.000 | 2.578 | 2.546 | 2.61 |
| Pre-existing hypertension | 0.294 | 0.025 | 135.115 | 1 | 0.000 | 1.342 | 1.277 | 1.411 |
| Remoteness |  |  | 51.547 | 3 | 0.000 |  |  |  |
| Inner Regional | 0.005 | 0.007 | 0.482 | 1 | 0.487 | 1.005 | 0.991 | 1.019 |
| Outer Regional | 0.058 | 0.012 | 24.279 | 1 | 0.000 | 1.06 | 1.036 | 1.084 |
| Remote/very remote | 0.152 | 0.028 | 29.445 | 1 | 0.000 | 1.165 | 1.102 | 1.23 |
| Hospital sector | -0.211 | 0.007 | 867.711 | 1 | 0.000 | 0.81 | 0.799 | 0.821 |
| Constant | -1.741 | 0.005 | 108343.5 | 1 | 0.000 | 0.175 |  |  |

| **Model 4: Dependent variable - Birthweight<2500 g** | | | | | | | | |
| --- | --- | --- | --- | --- | --- | --- | --- | --- |
|  | B | S.E. | Wald | df | Sig. | Exp(B) | 95% C.I.for EXP(B) | |
|  |  |  |  |  |  |  | Lower | Upper |
| Breast Cancer During Pregnancy | 2.209 | 0.211 | 109.658 | 1 | 0.000 | 9.104 | 6.022 | 13.765 |
| Country of birth | -0.094 | 0.009 | 113.578 | 1 | 0.000 | 0.91 | 0.894 | 0.926 |
| Maternal age | 0.269 | 0.01 | 776.48 | 1 | 0.000 | 1.308 | 1.284 | 1.333 |
| Parity | -0.425 | 0.008 | 3073.666 | 1 | 0.000 | 0.653 | 0.644 | 0.663 |
| Smoking during pregnancy | 0.933 | 0.009 | 11485.83 | 1 | 0.000 | 2.542 | 2.499 | 2.586 |
| Pre-existing hypertension | 1.066 | 0.026 | 1628.446 | 1 | 0.000 | 2.904 | 2.757 | 3.058 |
| Remoteness |  |  | 98.679 | 3 | 0.000 |  |  |  |
| Inner Regional | -0.083 | 0.01 | 64.87 | 1 | 0.000 | 0.921 | 0.902 | 0.939 |
| Outer Regional | -0.106 | 0.017 | 38.331 | 1 | 0.000 | 0.899 | 0.869 | 0.93 |
| Remote/very remote | 0.09 | 0.039 | 5.339 | 1 | 0.021 | 1.094 | 1.014 | 1.181 |
| Hospital sector | -0.639 | 0.012 | 2847.962 | 1 | 0.000 | 0.528 | 0.516 | 0.54 |
| Constant | -2.932 | 0.008 | 125685.5 | 1 | 0.000 | 0.053 |  |  |

| **Model 5: Dependent variable - 5 min Apgar 7 or less** | | | | | | | | |
| --- | --- | --- | --- | --- | --- | --- | --- | --- |
|  | B | S.E. | Wald | df | Sig. | Exp(B) | 95% C.I.for EXP(B) | |
|  |  |  |  |  |  |  | Lower | Upper |
| Breast Cancer During Pregnancy | 0.277 | 0.458 | 0.364 | 1 | 0.546 | 1.319 | 0.537 | 3.239 |
| Country of birth | 0.105 | 0.011 | 98.906 | 1 | 0.000 | 1.111 | 1.088 | 1.135 |
| Maternal age | 0.139 | 0.012 | 143.366 | 1 | 0.000 | 1.149 | 1.123 | 1.175 |
| Parity | -0.494 | 0.009 | 2982.286 | 1 | 0.000 | 0.61 | 0.599 | 0.621 |
| Smoking during pregnancy | 0.176 | 0.012 | 222.12 | 1 | 0.000 | 1.192 | 1.165 | 1.22 |
| Pre-existing hypertension | 0.417 | 0.038 | 120.63 | 1 | 0.000 | 1.518 | 1.409 | 1.635 |
| Remoteness |  |  | 80.358 | 3 | 0.000 |  |  |  |
| Inner Regional | 0.088 | 0.012 | 55.897 | 1 | 0.000 | 1.092 | 1.067 | 1.118 |
| Outer Regional | 0.047 | 0.02 | 5.472 | 1 | 0.019 | 1.048 | 1.008 | 1.089 |
| Remote/very remote | 0.251 | 0.046 | 29.707 | 1 | 0.000 | 1.286 | 1.175 | 1.407 |
| Hospital sector | -0.608 | 0.013 | 2114.893 | 1 | 0.000 | 0.544 | 0.53 | 0.559 |
| Mode of birth | 0.413 | 0.01 | 1842.137 | 1 | 0.000 | 1.512 | 1.484 | 1.541 |
| Constant | -3.351 | 0.01 | 107099.6 | 1 | 0.000 | 0.035 |  |  |

| **Model 6: Dependent variable - High Resuscitation** | | | | | | | | |
| --- | --- | --- | --- | --- | --- | --- | --- | --- |
|  | B | S.E. | Wald | df | Sig. | Exp(B) | 95% C.I.for EXP(B) | |
|  |  |  |  |  |  |  | Lower | Upper |
| Breast Cancer During Pregnancy | 0.698 | 0.301 | 5.386 | 1 | 0.020 | 2.009 | 1.115 | 3.621 |
| Country of birth | 0.169 | 0.008 | 408.092 | 1 | 0.000 | 1.185 | 1.165 | 1.204 |
| Maternal age | 0.114 | 0.009 | 165.489 | 1 | 0.000 | 1.12 | 1.101 | 1.14 |
| Parity | -0.432 | 0.007 | 3692.655 | 1 | 0.000 | 0.649 | 0.64 | 0.658 |
| Smoking during pregnancy | 0.151 | 0.01 | 236.574 | 1 | 0.000 | 1.163 | 1.141 | 1.185 |
| Pre-existing hypertension | 0.454 | 0.029 | 241.225 | 1 | 0.000 | 1.575 | 1.487 | 1.667 |
| Remoteness |  |  | 330.699 | 3 | 0.000 |  |  |  |
| Inner Regional | 0.156 | 0.009 | 280.324 | 1 | 0.000 | 1.168 | 1.147 | 1.19 |
| Outer Regional | 0.136 | 0.016 | 75.228 | 1 | 0.000 | 1.146 | 1.111 | 1.182 |
| Remote/very remote | 0.221 | 0.039 | 31.551 | 1 | 0.000 | 1.247 | 1.155 | 1.347 |
| Hospital sector | -0.449 | 0.01 | 2211.199 | 1 | 0.000 | 0.638 | 0.627 | 0.65 |
| Mode of birth | 0.699 | 0.007 | 9269.303 | 1 | 0.000 | 2.011 | 1.983 | 2.04 |
| Constant | -2.782 | 0.008 | 112451.3 | 1 | 0.000 | 0.062 |  |  |

| **Model 7: Dependent variable - admitted to NICU/SCN** | | | | | | | | |
| --- | --- | --- | --- | --- | --- | --- | --- | --- |
|  | B | S.E. | Wald | df | Sig. | Exp(B) | 95% C.I.for EXP(B) | |
|  |  |  |  |  |  |  | Lower | Upper |
| Breast Cancer During Pregnancy | 1.319 | 0.19 | 48.168 | 1 | 0.000 | 3.74 | 2.577 | 5.429 |
| Country of birth | 0.01 | 0.005 | 3.883 | 1 | 0.049 | 1.01 | 1 | 1.02 |
| Maternal age | 0.121 | 0.006 | 472.446 | 1 | 0.000 | 1.129 | 1.116 | 1.141 |
| Parity | -0.33 | 0.004 | 5574.624 | 1 | 0.000 | 0.719 | 0.713 | 0.725 |
| Smoking during pregnancy | 0.372 | 0.006 | 4157.48 | 1 | 0.000 | 1.451 | 1.435 | 1.467 |
| Pre-existing hypertension | 0.663 | 0.019 | 1240.315 | 1 | 0.000 | 1.94 | 1.87 | 2.013 |
| Remoteness |  |  | 701.215 | 3 | 0.000 |  |  |  |
| Inner Regional | -0.121 | 0.006 | 399.663 | 1 | 0.000 | 0.886 | 0.876 | 0.897 |
| Outer Regional | -0.208 | 0.01 | 400.204 | 1 | 0.000 | 0.812 | 0.795 | 0.829 |
| Remote/very remote | 0.03 | 0.025 | 1.529 | 1 | 0.216 | 1.031 | 0.982 | 1.082 |
| Hospital sector | -0.55 | 0.006 | 8250.663 | 1 | 0.000 | 0.577 | 0.57 | 0.584 |
| Mode of birth | 0.786 | 0.005 | 28853.65 | 1 | 0.000 | 2.194 | 2.175 | 2.214 |
| Constant | -1.86 | 0.008 | 53047.27 | 1 | 0.000 | 0.156 |  |  |

**Goodness of fit tests**

| **Model 1: Hosmer and Lemeshow Test** | | | |  | **Model 2: Hosmer and Lemeshow Test** | | | |  | **Model 3: Hosmer and Lemeshow Test** | | | |
| --- | --- | --- | --- | --- | --- | --- | --- | --- | --- | --- | --- | --- | --- |
| Step | Chi-square | df | Sig. |  | Step | Chi-square | df | Sig. |  | Step | Chi-square | df | Sig. |
| 1 | 3.345 | 7 | 0.851 |  | 1 | 102.135 | 8 | 0.000 |  | 1 | 1567.373 | 8 | 0.000 |
|  |  |  |  |  |  |  |  |  |  |  |  |  |  |
| **Model 4: Hosmer and Lemeshow Test** | | | |  | **Model 5: Hosmer and Lemeshow Test** | | | |  | **Model 6: Hosmer and Lemeshow Test** | | | |
| Step | Chi-square | df | Sig. |  | Step | Chi-square | df | Sig. |  | Step | Chi-square | df | Sig. |
| 1 | 638.879 | 8 | 0.000 |  | 1 | 238.685 | 7 | 0.000 |  | 1 | 395.891 | 8 | 0.000 |
|  |  |  |  |  |  |  |  |  |  |  |  |  |  |
| **Model 7: Hosmer and Lemeshow Test** | | | |  |  |  |  |  |  |  |  |  |  |
| Step | Chi-square | df | Sig. |  |  |  |  |  |  |  |  |  |  |
| 1 | 579.617 | 8 | 0.000 |  |  |  |  |  |  |  |  |  |  |

The sample size in this study is large, comprising a total of 1,783,116 women. Results from Hosmer and Lemeshow test like other chi-square tests are highly affected by the sample size, and when the sample size is large the test may indicate a poor fit even with a minimal deviation between observed and predicted values(1-3). Therefore it was suggested that Hosmer and Lemeshow test will not work when the sample size excceds 25,000 and it its also sensitive to the number of groups (1,2).

1. Yu W, Xu W, Zhu L. A modified Hosmer–Lemeshow test for large data sets. Communications in Statistics-Theory and Methods. 2017 Dec 2;46(23):11813-25.
2. Lai X, Liu L. A simple test procedure in standardizing the power of Hosmer–Lemeshow test in large data sets. Journal of Statistical Computation and Simulation. 2018 Sep 2;88(13):2463-72.
3. Paul P, Pennell ML, Lemeshow S. Standardizing the power of the Hosmer–Lemeshow goodness of fit test in large data sets. Statistics in medicine. 2013 Jan 15;32(1):67-80.
